# Prognostic value of prostaglandin I2 synthase and its correlation with tumor-infiltrating immune cells in lung cancer, ovarian cancer, and gastric cancer

**DOI:** 10.18632/aging.103235

**Published:** 2020-05-28

**Authors:** Danian Dai, Bo Chen, Yanling Feng, Weizhong Wang, Yanhui Jiang, He Huang, Jihong Liu

**Affiliations:** 1 Department of Gynecology and Obstetrics, The Fifth Affiliated Hospital of Sun Yat-Sen University, Zhuhai 519000, Guangdong, China; 2 Department of Gynecologic Oncology, State Key Laboratory of Oncology in South China, Collaborative Innovation Center for Cancer Medicine, Sun Yat-Sen University Cancer Center, Guangzhou 510060, Guangdong, China; 3 Department of Breast Cancer, Cancer Center, Guangdong Provincial People's Hospital and Guangdong Academy of Medical Sciences, Guangzhou 510080, Guangdong, China; 4 Department of Respiratory Medicine, The First Affiliated Hospital of University of South China, Hengyang 421001, Hunan, China

**Keywords:** prostaglandin I2 synthase, tumor-associated macrophages, prognosis, lung cancer, ovarian cancer

## Abstract

Background: Prostaglandin I2 synthase (PTGIS) is a crucial gene for the synthesis of prostaglandin I2, which has multiple roles in inflammation and immune modulation. However, studies on the prognostic value of PTGIS and its correlation with tumor-infiltrating immune cells in multiple cancers are still rare.

Results: Multiple datasets of the Oncomine database showed that PTGIS was expressed at low levels in lung cancer and ovarian cancer compared to the levels in normal tissues. Kaplan-Meier plotter showed that high PTGIS was associated with poor overall survival and progression-free survival in lung, ovarian, and gastric cancers. Moreover, PTGIS expression was significantly positively correlated with infiltrating levels of macrophages and was strongly associated with a variety of immune markers, especially tumor-associated macrophages (TAMs) and T-regulatory cells (Tregs).

Conclusions: High expression of PTGIS could promote the infiltration of TAMs and Tregs in the tumor microenvironment and deteriorate outcomes of patients with lung, ovarian, and gastric cancers. These findings suggest that PTGIS could be taken as a potential biomarker of prognosis and tumor-infiltrating immune cells.

Methods: PTGIS expression was investigated in different datasets of the Oncomine database, and its expression levels in various tumors and corresponding normal tissues were analyzed by the Tumor Immune Estimation Resource (TIMER). Then, the clinical prognostic value of PTGIS was assessed with online public databases. In addition, we initially explored the correlation between PTGIS and tumor-infiltrating immune cells by TIMER and Gene Expression Profiling Interactive Analysis (GEPIA).

## INTRODUCTION

Solid tumors are the most extensive and common malignant tumors worldwide, including lung tumors, ovarian tumors, and gastric tumors. Insidious onset, invasive and fast growth, and high recurrence and metastasis rates are common characteristics leading to poor prognosis [1]. Recently, immunotherapy has been widely used in the treatment of solid tumors, including melanoma and lung, ovarian, breast, and stomach cancers, and its tolerable toxicity and long-term survival improvement have benefited many advanced cancer patients, leaving immunotherapy as the most promising direction for curing cancer [2]. Some immunotherapies, such as programmed death-1 (PD-1) and programmed death ligand-1 (PD-L1) inhibitors or cytotoxic T lymphocyte-associated antigen 4 (CTLA4) therapies, have shown an optimistic antitumor effect in melanoma [3, 4], lung cancer [5], gastrointestinal cancer [6] and ovarian cancer [7]. However, the current anti-CTLA-4 agent showed no effect in a clinical study of prostate cancer [8], and anti-PD1 therapy showed less effect in colorectal cancer [9] and even promoted tumor progression for some patients with murine double minute2 (MDM2) amplification or epidermal growth factor receptor (EGFR) aberration [10]. Moreover, increasing evidence has demonstrated that tumor-infiltrating immune cells interact with tumor cells and immunotherapy and have important implications for efficacy and patient outcomes [11–13]. Therefore, the elucidation of the mechanism of the interaction between tumor phenotype and infiltrating immune cells in the microenvironment and the exploration of new immune-related therapeutic targets are urgent for the treatment of solid tumors.

Prostaglandin I2 synthase (PTGIS) is a protein-encoding gene localized on chromosome 20q13.11-q13.13 and was first reported in 1996 [14]. PTGIS encodes a member of the cytochrome P450 superfamily, a monooxygenase that catalyzes the metabolism of many drugs and the synthesis of lipids such as cholesterol and steroids. In addition, PTGIS could be involved in iron and heme metabolism, oxidative stress, xenobiotic and drug metabolism, glutathione and prostaglandin metabolism, and the conversion of prostaglandin H2 to prostaglandin I2 (PGI2) [14, 15]. A previous study observed that hypermethylation of the PTGIS promotor was associated with diminished gene expression in colorectal carcinogenesis [16]. Furthermore, other studies suggested that PTGIS variants may affect breast cancer susceptibility [17], and elevated PTGIS was associated with liver metastasis and poor survival outcomes for patients with colon cancer [18]. These findings suggest that PTGIS has distinctly essential impacts on tumorigenesis, progression, and metastasis.

PGI2 is an important product of the arachidonic acid (AA) metabolism pathway, and PTGIS is one of the key enzymes. PGI2 is involved in inflammatory responses and activation of CD4+ T cells during physiological processes [19]. In addition, PGI2 is a crucial immunoregulatory lipid mediator that affects the differentiation of Th17 cells and T-regulatory cells (Tregs) [20]. The above results suggest that PTGIS has an indirect regulatory effect on microenvironment immune cells. Nevertheless, the potential functions and mechanisms of PTGIS in tumorigenesis and development and the immune microenvironment are undefined.

In this study, our aim was to comprehensively analyze the relationship between the expression of PTGIS and prognosis in cancer patients and to explore the correlation between PTGIS and tumor-infiltrating immune cells. Our findings provide new ideas for elucidating the potential mechanism of PTGIS in tumor progression and the mechanism by which PTGIS is associated with tumor-infiltrating immune cells.

## RESULTS

### PTGIS expression level in various kinds of tumors

**Figure 1:**
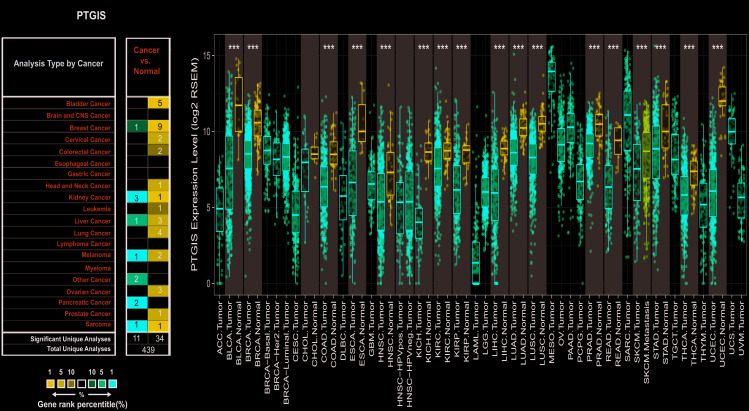
**Expression of PTGIS in various human tumors.** (**A**) Increased or decreased expression of PTGIS in different tumors compared to normal tissues in the Oncomine database. (**B**) PTGIS expression of different tumor types from the TCGA database was investigated by TIMER (*P < 0.05, **P < 0.01, ***P < 0.001).

To investigate the expression levels of PTGIS, the PTGIS mRNA levels in various tumors and normal samples were analyzed with the Oncomine database. Across various cancer types, significantly more datasets showed low expression of PTGIS in cancer samples versus normal samples than overexpression of PTGIS (Figure 1A). The expression of PTGIS was absolutely lower in bladder cancer, cervical cancer, colorectal cancer, head and neck cancer, leukemia, lung cancer, ovarian cancer, and prostate cancer than in normal samples. In addition, higher expression was found in pancreatic cancer and other cancer samples than in the corresponding normal samples, and the expression levels in some cancers were controversial. The specific data of PTGIS mRNA expression levels in various cancer datasets are displayed in Supplementary Table 1. Next, we further examined PTGIS expression in multiple human cancers with RNA-seq data from The Cancer Genome Atlas (TCGA). The expression levels of PTGIS between tumor and matched normal tissues in all TCGA datasets are shown in Figure 1B. Taken together, the data confirmed that the PTGIS gene was downregulated in multiple cancers compared to normal samples.

### Prognostic value of PTGIS in cancers

**Figure 2:**
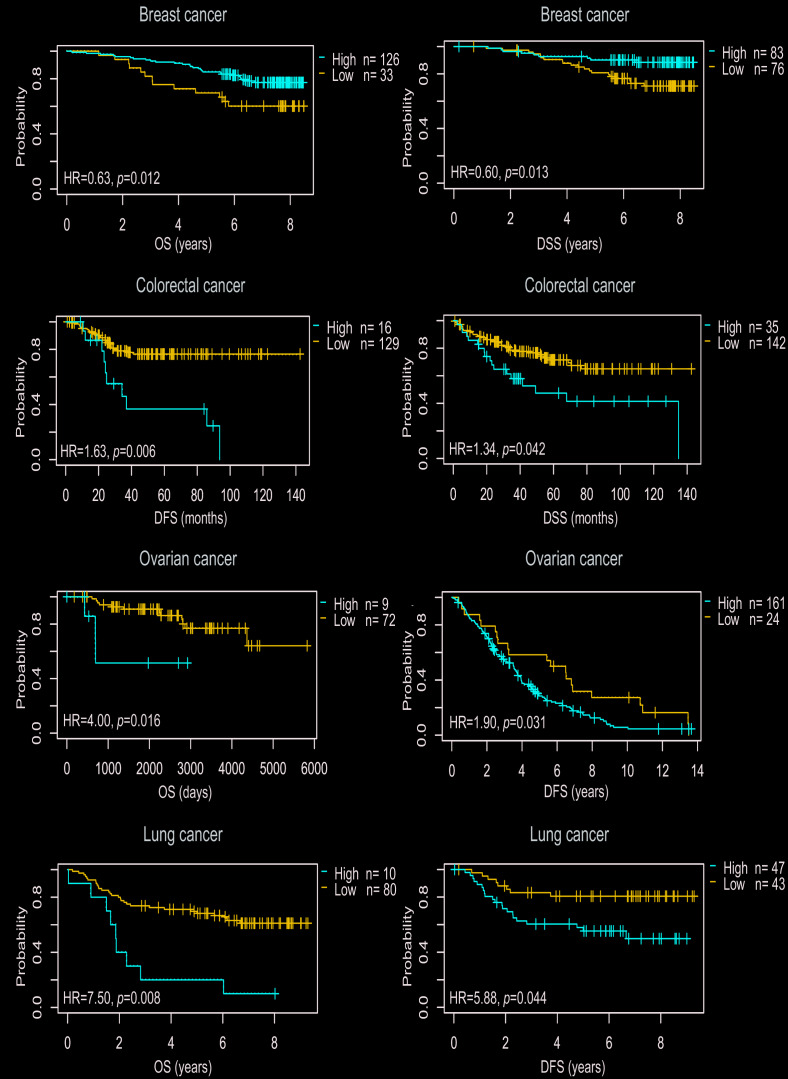
**Survival curves of high or low expression of PTGIS in different tumors from the PrognoScan database.** (**A**, **B**) High PTGIS expression was correlated with better OS and DSS than low PTGIS expression in the breast cancer cohort [GSE1456-GPL96 (n = 159)]. (**C**, **D**) High PTGIS expression was correlated with poorer DFS (n = 145) and DSS (n = 177) than low PTGIS expression in the colorectal cancer cohort (GSE17536). (**E**, **F**) High PTGIS expression was correlated with poorer OS and DFS than low PTGIS expression in two ovarian cancer cohorts [GSE8841 (n = 81) and GSE26712 (n = 185)]. (**G**, **H**) High PTGIS expression was correlated with poorer OS and DSS than low PTGIS expression in a lung cancer cohort (GSE14814, n = 90). OS, overall survival; DFS, disease-free survival; DSS, disease-specific survival.

To explore the correlation between PTGIS expression and prognosis in human cancers, we investigated the effects of PTGIS expression on survival via PrognoScan. Eight out of thirteen cancers showed a potential correlation between PTGIS and prognosis (Supplementary Table 2). Interestingly, compared with low PTGIS expression, high expression of PTGIS indicated a better survival prognosis for overall survival (OS) (hazard ratio [HR]=0.63, 95% confidence interval [CI]=0.44 to 0.90, P=0.012) and disease specific survival (DSS) (HR=0.60, 95% CI=0.40 to 0.90, P=0.013) in breast cancer (Figure 2A and 2B). However, among the other three common solid tumors (colorectal cancer, ovarian cancer, and lung cancer), high PTGIS expression was associated with a worse prognosis than low PTGIS expression (Figure 2C–2H). In addition, PTGIS had no significant effect on OS in colorectal cancer. The survival plots generated from different datasets are shown in Supplementary Figure 2.

**Figure 3:**
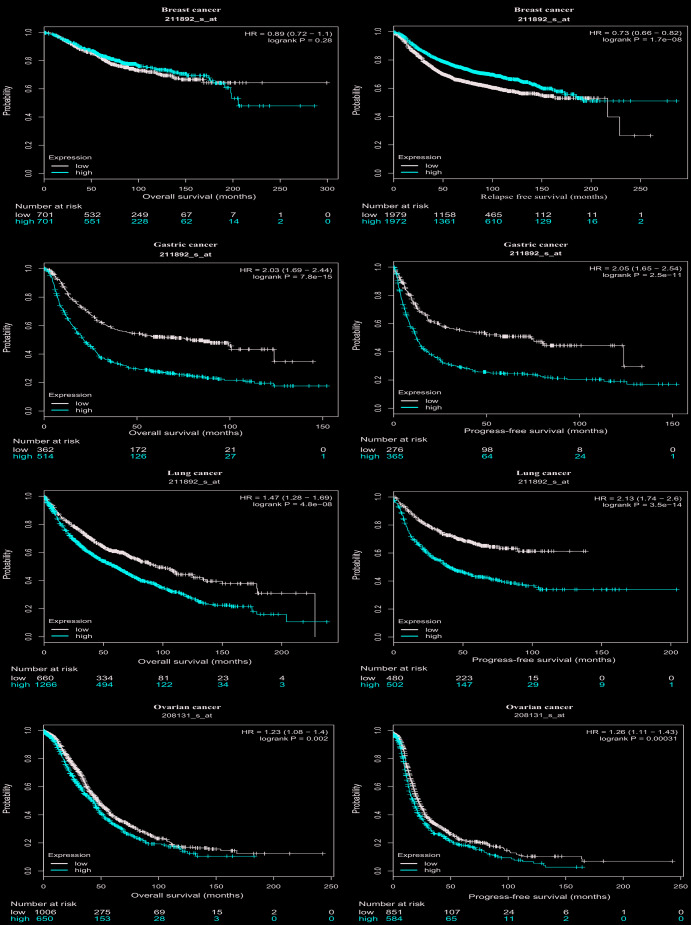
**Survival curves of high or low expression of PTGIS in different tumors from Kaplan-Meier plotter.** (**A**, **B**) OS and DFS survival curves of breast cancer (n = 1,402 and n = 3,951, respectively). (**C**, **D**) OS and PFS survival curves of gastric cancer (n = 876 and n = 641, respectively). (**E**, **F**) OS and PFS survival curves of lung cancer (n = 1,926 and n = 982, respectively). (**G**, **H**) OS and PFS survival curves of ovarian cancer (n = 1,656 and n = 1,435, respectively). OS, overall survival; PFS, progression-free survival; DFS, disease-free survival.

Then, we further assessed the prognostic value of PTGIS in tumors with the Kaplan-Meier plotter, which is based on Affymetrix microarray data. Notably, PTGIS had less influence on OS in this analysis than it had been shown to have in the PrognoScan analysis for breast cancer (HR=0.89, 95% CI=0.72 to 1.1, P=0.28) (Figure 3A), and high PTGIS expression was correlated with poor prognosis in gastric cancer (OS HR=2.03, 95% CI=1.69 to 2.44, P=7.8e-15; progression-free survival [PFS] HR=2.05, 95% CI=1.65 to 2.54, P=2.5e-11) (Figure 3C and 3D). Consistent with previous results, patients with high expression of PTGIS had a poor prognosis in both lung cancer (OS HR=1.47, 95% CI=1.28 to 1.69, P=4.8e-08; PFS HR=2.13, 95% CI=1.74 to 2.6, P=3.5e-14) and ovarian cancer (OS HR=1.23, 95% CI=1.08 to 1.4, P=0.002; PFS HR=1.26, 95% CI=1.11 to 1.43, P=3.1e-4) (Figure 3E–3H). Based on this large-sample validation analysis, these results suggest that high PTGIS expression implies reduced survival in ovarian, lung and gastric cancer.

The above analyses of PTGIS were based on microarray data from Kaplan-Meier plotter and the PrognoScan database. The prognostic value of PTGIS was explored for various tumors with RNA-seq data from TCGA with the Gene Expression Profiling Interactive Analysis (GEPIA) website. A total of 33 cancer types were included in the analysis of the relationship between PTGIS expression and survival (Supplementary Figure 3). Compared with downregulated PTGIS expression, elevated PTGIS expression was associated with worse OS or disease free survival (DFS) in ACC (adrenocortical carcinoma), BLCA (bladder urothelial carcinoma), COAD (colon adenocarcinoma), GBM (glioblastoma multiforme), KIRP (kidney renal papillary cell carcinoma), LUSC (lung squamous cell carcinoma), OV (ovarian serous cystadenocarcinoma) and STAD (stomach adenocarcinoma). In addition, elevated PTGIS expression was associated with improved DFS for only SARC (sarcoma). These results demonstrate the important prognostic value of PTGIS as an oncogene in certain types of cancer, suggesting that it plays a crucial role in the progression of cancer.

### High expression of PTGIS deteriorates the outcomes of ovarian and gastric cancer patients with lymph node metastasis

To explore the potential mechanism by which PTGIS expression affects prognosis, we studied the association between expression levels of PTGIS and clinical variables in ovarian (Supplementary Table 3) and gastric cancer patients (Supplementary Table 4). For serous ovarian cancer, high expression of PTGIS was related to reduced OS and PFS. Specifically, compared with low PTGIS mRNA expression, high PTGIS mRNA expression was correlated with worse OS and PFS only in stage 3 disease (OS HR = 1.2, P = 0.0398; PFS HR = 1.28, P = 0.0025), which includes involvement of retroperitoneal lymph nodes [21]. In addition, PTGIS high expression alone did not impair the OS of patients treated with optimal debulking surgery. For gastric cancer patients, compared with lower levels of PTGIS, elevated PTGIS was correlated with worse OS and PFS after stratification by HER2 status, Lauren classification, or differentiation (P < 0.05). Moreover, PTGIS expression had a significant prognostic correlation with the N stage. Stages N0-4 indicate different degrees of regional lymph node metastasis [22]. The above results imply that the expression level of PTGIS can deteriorate the prognosis of patients with ovarian or gastric cancer with lymph node metastasis.

### The expression level of PTGIS is positively correlated with infiltrating immune cells in lung, ovarian and gastric cancers

**Figure 4:**
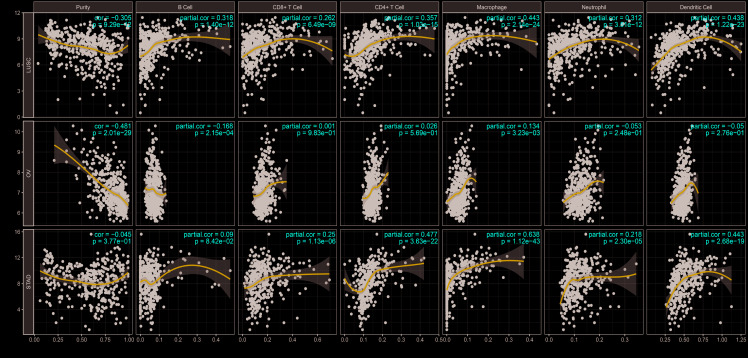
**Correlation of PTGIS expression with immune infiltration level in LUSC (lung squamous cell carcinoma), OV (ovarian serous cystadenocarcinoma) and STAD (stomach adenocarcinoma).** (**A**) PTGIS expression was significantly negatively related to tumor purity and had significant positive correlations with the levels of infiltrating B cells, CD8+ T cells, CD4+ T cells, macrophages, neutrophils, and dendritic cells in LUSC (n = 496). (**B**) PTGIS expression was significantly negatively related to tumor purity and the levels of infiltrating B cells but has no significant correlations with the levels of infiltrating CD8+ T cells, CD4+ T cells, neutrophils, and dendritic cells in OV. PTGIS expression showed a very weak positive correlation with macrophage infiltration in OV (n = 537). (**C**) PTGIS expression had no significant correlations with tumor purity and the levels of infiltrating B cells but had significant positive correlations with the levels of infiltrating CD8+ T cells, CD4+ T cells, macrophages, neutrophils, and dendritic cells in STAD (n= 407).

Tumor-infiltrating lymphocytes (TILs) are associated with sentinel lymph node metastasis and prognosis in tumors [23–25]. Thus, the correlation between PTGIS and tumor-infiltrating immune cells was assessed in different cancers with TIMER. We observed that PTGIS expression levels were significantly associated with tumor purity in 26 kinds of cancer, of which 23 kinds of cancer showed a negative correlation between PTGIS expression and tumor purity. In addition, PTGIS expression was significantly correlated with infiltrating immune cells, including B cells, CD4+/CD8+ T cells, macrophages, neutrophils, and dendritic cells, in various types of cancers (Figure 4 and Supplementary Figure 4). After the preliminary analysis of the correlation between PTGIS and infiltrating immune cells in various cancers, we then selected the specific cancers in which PTGIS was correlated with oncologic outcomes and infiltrating immune cells. It was reported that the tumor purity level had an impact on immune infiltration in an analysis of clinical sample data based on genetic testing [26, 27]. TIMER and GEPIA have most of the common transcriptomics data derived from the TCGA database [28, 29], so we selected the types of cancer in TIMER in which PTGIS had a significantly negative correlation with tumor purity and prognostic significance in GEPIA. Based on the prognostic results related to PTGIS from the PrognoScan, Kaplan-Meier-plotter and GEPIA analyses, we eventually selected LUSC, OV and STAD for further research on immune infiltration via TIMER. The PTGIS expression level had a significant negative correlation with tumor purity but significant positive correlations with the levels of 6 infiltrating immune cells in LUSC (Figure 4A). However, there were significantly negative correlations with tumor purity (r = -0.481, P = 2.01e-29) and the level of infiltrating B cells (r = -0.168, P = 2.15e-04) and a positive correlation with only macrophages (r = 0.134, P = 3.23e-03) in OV (Figure 4B). Interestingly, there was no significant correlation with tumor purity (r = -0.045, P = 3.77e-01) and the level of infiltrating B cells (r = -0.09, P = 8.42e-02) but significant positive correlations with the levels of infiltrating CD8+ T cells (r = 0.25, P = 1.13e-06), CD4+ T cells (r = 0.477, P =3.63e-22), macrophages (r = 0.638, P = 1.12e-43), neutrophils (r = 0.218, P = 2.30e-05), and DCs (r = 0.443, P = 2.68e-19) in STAD (Figure 4C). These findings strongly demonstrate that PTGIS could recruit immune cells in the tumor microenvironment (TME) in LUSC, OV and STAD, especially on macrophages.

### Correlation analysis between PTGIS and markers of infiltrating immune cells

**Figure 5:**
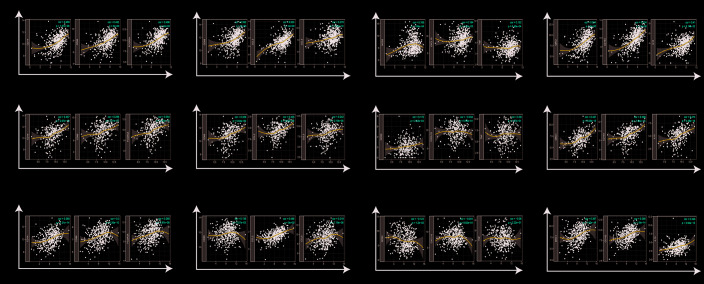
**PTGIS expression correlated with macrophage polarization in LUSC (lung squamous cell carcinoma), OV (ovarian serous cystadenocarcinoma) and STAD (stomach adenocarcinoma).** Markers included CD14, CD86 and FCGR3A for monocytes; CD68, CCL2 and CCL5 for TAMs (tumor-associated macrophages); NOS2, CXCL10, and TNF for M1 macrophages; and MRC1, CD163, and IL10 for M2 macrophages. (**A**–**D**) Scatterplots of correlation between PTGIS expression and the expression of gene markers of monocytes (A), TAMs (**B**), and M1 (**C**) and M2 macrophages (**D**) in LUSC (n = 496). (**E**–**H**) Scatterplots of correlation between PTGIS expression and the expression of gene markers of monocytes (**E**), TAMs (**F**), and M1 (**G**) and M2 macrophages (**H**) in OV (n = 537). (**I**–**L**) Scatterplots of correlation between PTGIS expression and the expression of gene markers of monocytes (**A**), TAMs (**B**), and M1 (**C**) and M2 macrophages (**D**) in STAD (n= 407).

To explore the effects of PTGIS expression on tumor-infiltrating immune cells, we analyzed the relationships between PTGIS expression and various markers of immune cells in LUSC, OV, and STAD via public databases. We selected some of the infiltrating immune cells, including innate immune cells (Supplementary Table 5) and adaptive immune cells (Supplementary Table 6), and analyzed the relationship between PTGIS and specific markers of these immune cells in LUSC, OV and STAD (Figure 5). In LUSC and STAD, the changes in correlation coefficients between the expression level of PTGIS and the expression of gene marker sets of different immune cells were not significant after adjustment for purity. However, the association between PTGIS and immune markers changed dramatically in OV. It is worth noting that the correlation between PTGIS and various immune cell markers was significantly increased without adjustment for purity.

Specifically, we found that PTGIS expression was more highly correlated with gene markers of monocytes/macrophages (monocytes, TAMs, and M2 macrophages) than with gene markers of other infiltrating immune cells in LUSC, OV and STAD (Supplementary Table 5). In addition, we determined the correlation coefficients between PTGIS and specific markers of monocytes, TAMs, M1 macrophages, and M2 macrophages in LUSC, OV and STAD (Figure 5). We further investigated the relationship between PTGIS and the above gene markers of immune cells in normal tissues and tumors using the GEPIA database. Notably, there was no correlation between PTGIS and most immune markers of monocytes and TAMs in normal lung tissues. The results of the correlation were generally consistent with those of the TIMER analysis in tumors (Supplementary Table 7). These results imply that PTGIS likely plays a promoting role in the regulation of macrophage polarization in LUSC, OV, and STAD.

Elevated PTGIS expression levels were associated with a high degree of Tregs infiltration in LUSC, OV and STAD, and Tregs markers such as FOXP3, STAT5B, TGFB1, and IL2RA also showed obvious correlations with PTGIS expression (Supplementary Table 6). These results suggest a strong positive correlation between PTGIS and Tregs infiltration. There is evidence that Tregs can negatively regulate CD8+ T cell and natural killer cell responses to tumor cells as well as promote angiogenesis and metastasis [30]. Whether PTGIS is a pivotal factor that activates Tregs and tumor progression still needs further study.

Furthermore, we also observed a significant positive correlation between PTGIS and some of the markers of Tregs and T cell exhaustion, including FOXP3, STAT5B, TGFB1 (TGFβ), IL2RA (CD25), and HAVCR2 (TIM-3), in LUSC, OV, and STAD. FOXP3 has a crucial role in the development and function of Tregs, and excessive Tregs could prevent the immune system from destroying cancer cells and promote cancer progression [31]. Interestingly, PTGIS expression also has a positive correlation with TIM-3, an important gene mediating T cell exhaustion and macrophage activation; the presence of the exhausted phenotype downregulates the immune response in tumor-bearing hosts [32, 33]. Therefore, these results further confirm the correlation between PTGIS and infiltrating immune cells in the microenvironment of LUSC, OV, and STAD and indicate that PTGIS promotes significantly to the process of tumor immune escape.

## DISCUSSION

PTGIS is a member of the P450 superfamily and a membrane protein that localizes to the endoplasmic reticulum. It is widely expressed in various tissues, especially in the lung, ovary, skeletal muscle and prostate. The main product of this enzyme is PGI2, which is the major metabolite of AA and a potent vasodilator and platelet aggregation inhibitor [14]. Although studies of PTGIS are still few, it is known that PTGIS may play an important role in tumorigenesis and cancer development in colon cancer, lung cancer, breast cancer, and head and neck cancer [17, 18, 34, 35]. In addition, PGI2, as a product of PTGIS, has a pro-inflammatory effect that increases microvascular permeability and an anti-inflammatory effect that stimulates T cell IL-10 production [36]. Furthermore, it was reported that PGI2 signaling could increase immature dendritic cell migration and inhibit immune responses [37]. In our study, we observed that the PTGIS expression level was associated with the prognosis of various cancers. Compared with low PTGIS expression, elevated PTGIS was associated with a poorer outcome in LUSC, OV, and STAD. Notably, high expression of PTGIS could significantly impair the prognosis of patients with lymph node metastasis in ovarian or gastric cancer. In addition, our further analysis showed that immune cell infiltration levels and various immunological markers were associated with PTGIS expression levels in LUSC, OV, and STAD. Therefore, our study provides clues to shed light on the potential effects of PTGIS in the TME and its application as a prognostic biomarker.

In our research, PTGIS mRNA expression profiles and prognosis were analyzed with datasets from multiple kinds of cancer from Oncomine and TCGA. In the comparison of various cancers with normal tissues, we observed differences in PTGIS expression. According to the analysis of the Oncomine data, PTGIS showed low expression in most tumors compared to that in normal tissues, and the TCGA data confirmed these results in BLCA, BRCA, COAD, ESCA, HNSC, KICH, KIRC, KIRP, LIHC, LUAD, LUSC, PRAD, READ, SKCM, STAD, THCA and UCEC (Figure 1A and 1B). It has been reported in the literature that hypermethylation of gene promoters leads to transcriptional silencing as a common event in cancer, and hypermethylation of the PTGIS promoter was also detected in colorectal cancer [16]. Because of the differences in data collection and processing mechanisms between different databases, the expression of and prognosis related to PTGIS may be inconsistent in these data. For example, high expression of PTGIS was associated with a good prognosis for breast cancer patients in PrognoScan, while there was no significant effect on prognosis in Kaplan-Meier-plotter and the GEPIA database. However, in these databases, we found consistent results regarding prognosis in lung, ovarian, and gastric cancers (Figure 3 and Supplementary Figure 3). Moreover, compared with low expression of PTGIS, elevated expression of PTGIS was revealed to be associated with poorer survival outcomes for patients with stage 3 disease, patients with wild-type TP53, and patients treated with suboptimal debulking surgery in ovarian cancer, as well as for patients with advanced-stage disease or lymph node metastasis in gastric cancer. In summary, these results powerfully demonstrate that PTGIS is a prognostic marker for lung, ovarian, and gastric cancers.

We found that PTGIS expression was associated with tumor immune cell infiltration in lung, ovarian, and gastric cancers. It was reported that the types of tumor-infiltrating immune cells could be determined from statistical approaches using tumor RNA-seq data of a series of immune cell-specific genes [38]. However, tumor purity can confuse such genomic sequencing analyses, and thus, coexpression analysis should use partial correlation analysis to adjust for tumor purity [39]. After purity adjustment, we found that the correlation of genes obviously changed, especially the values in ovarian cancer, which were the most significant changes (Supplementary Tables 5 and 6). Interestingly, immune-specific genes of M1 macrophages, such as NOS2, CXCL10, and TNF, displayed weak or no correlations with PTGIS, but M2 macrophage genes, such as MRC1, CD163, and IL10, displayed relatively strong correlations (Supplementary Tables 5 and 7). These findings suggest a possible activating effect of PTGIS in the polarization of TAMs. Moreover, our other findings imply that PTGIS also influences Tregs activation and induces T cell exhaustion to some extent. Increased expression of PTGIS was positively correlated with the expression of Tregs and T cell exhaustion markers (Supplementary Table 6). These correlations may indicate a potential mechanism by which PTGIS suppresses T cell function in LUSC, OV, and STAD. Therefore, the above results show that PTGIS plays a vital role in infiltrating immune cell recruitment and functional suppression in the TME.

Previous studies have provided possible explanations for why PTGIS expression in a tumor is associated with immune infiltration and poor prognosis. Platelets are the "first responders" to cancer and metastasis, and this initial role of platelets depends on the metabolism of prostacyclins; in addition, pharmacological, clinical, and epidemiological studies indicate that nonsteroidal anti-inflammatory drugs (NSAIDs), which target cyclooxygenases, could help prevent cancer [40]. PGI2 is the primary metabolite of PTGIS, and the 5-year survival rate of lung cancer patients with high expression of PGI2 is significantly worse than that of lung cancer patients with low expression of PGI2 [41]. PGI2, as a precursor of protumorigenic metabolites, not only promotes cancer growth by activating peroxisome proliferator-activated receptor δ (PPARδ) and increases the expression levels of the proangiogenic factor vascular endothelial growth factor [42] but also seems to act primarily on TAMs, which promote all aspects of cancer growth and progression [43]. PTGIS may be a crucial factor leading to increased accumulation of PGI2 in tumors and may affect the release of inflammatory factors through the synergistic action of the AA pathway, leading to the recruitment of various immune cells in the TME. PGI2 could regulate the innate immune system, including dendritic cells, macrophages, and monocytes, by increasing anti-inflammatory IL-10 and decreasing TNF-a, IL-1a, IL-6, and IL-12 [44]. Additionally, PGI2 displays an immunosuppressive capability via elevation of cAMP levels and downregulation of NF-kB [45]. The release cytokines and growth factors into the TME are crucial for tumor progression. Thus, the interaction between the AA pathway and the TME may be a likely reason explaining why elevated PTGIS leads to poorer outcomes in LUSC, OV, and STAD.

There are some limitations in this study. Since our study is based on data from public databases, it may have biases resulting from confounding factors. Moreover, the mechanisms by which PTGIS polarizes M1 macrophages into M2 macrophages are also unclear and need to be uncovered in future studies.

Our results showed that, compared with low PTGIS, elevated PTGIS suggested worse survival outcomes and promoted immune cell infiltration in diverse tumors. In addition, in lung, ovarian, and gastric cancers, the PTGIS expression level was closely related to the activation of immune cells, especially TAMs and Tregs, as well as T cell exhaustion. Thus, PTGIS may play a role of immune suppression by affecting tumor-infiltrating immune cells and be used as a prognostic marker for lung, ovarian and gastric cancer patients.

## MATERIALS AND METHODS

### Oncomine database analysis

PTGIS expression levels in different tumors were analyzed via the Oncomine database (https://www.oncomine.org) [46, 47]. The threshold settings were as follows: gene ranking of top 10%, fold change of 2.0, and P-value of 1E-4.

### PrognoScan database analysis

PrognoScan (http://dna00.bio.kyutech.ac.jp/PrognoScan/) [48] is a powerful platform that contains a great number of publicly available cancer microarray datasets with corresponding clinical information and is also a tool for assessing the biological relationship between gene expression and clinical outcomes. The associations between PTGIS expression levels and different cancer patient prognoses were obtained from the PrognoScan database. The threshold was specified as a P-value < 0.05.

### Kaplan-Meier-plotter database analysis

Kaplan-Meier plotter was used to analyze the association of PTGIS expression with prognosis in 5,353 breast, 3,091 ovarian, 2,909 lung, and 1,517 gastric cancer patients (http://kmplot.com/analysis/) [49]. The number of patients at risk at certain time points between subgroups based on gene expression status is provided in Kaplan-Meier survival plots. The hazard ratio (HRs), 95% confidence intervals (CIs) and log-rank P-values were calculated. A P-value <0.05 was considered statistically significant.

### TIMER database analysis

Tumor Immune Estimation Resource (TIMER) is a powerful computational tool for the systematic analysis of immune cell infiltration according to RNA sequencing data from various tumors (https://cistrome.shinyapps.io/timer/) [28, 50]. The expression of PTGIS in various cancers and its correlation with the abundances of six tumor-infiltrating immune cells (TIICs) (B cells, CD4+ T cells, CD8+ T cells, macrophages, neutrophils, and dendritic cells) was analyzed by the corresponding functional modules. According to related references [51–53] and the CellMarker database (http://biocc.hrbmu.edu.cn/CellMarker/) [54], a total of 66 related gene markers of TIICs were used for the analysis. The expression scatter plots between PTGIS and immune-related gene markers based on a specific cancer type were generated by correlation modules, and Spearman's correlation coefficient and the P-value are displayed. Gene expression levels are shown as log2 RSEM values.

### Gene correlation analysis in GEPIA

There is a new interactive web server for analyzing and visualizing RNA sequencing expression data called Gene Expression Profiling Interactive Analysis (GEPIA) (http://gepia.cancer-pku.cn/index.html) [29]. GEPIA is based on data from 9,736 tumors and 8,587 normal tissues from TCGA [55] and the Genotype-Tissue Expression (GTEx) Project [56], which was used to confirm the gene correlation analysis in TIMER. The survival plots of 33 different types of cancer were analyzed by GEPIA depending on the expression levels of a gene with the log-rank test. Gene expression correlation analysis was performed on tumor tissues and normal tissues using TCGA and GTEx datasets. The correlation coefficient was calculated by the Spearman method. PTGIS expression is displayed on the x-axis, and the expression of other genes is shown on the y-axis.

### Statistical methods

PrognoScan and Kaplan-Meier plots were used to obtain curves related to survival outcomes, including overall survival (OS), disease-free survival (DFS), and disease-specific survival (DSS). Gene expression profiling results from Oncomine are shown with gene rankings, fold changes, and P-values. All the data were from Kaplan-Meier plotter, PrognoScan, and GEPIA, and the results are displayed with P-values based on a log-rank test and a hazard ratio (HR). Spearman's correlation coefficients and P-values were used to evaluate gene correlation. P-values less than 0.05 were considered statistically significant. The flow diagram is displayed in Supplementary Figure 1.

## Supplementary Material

Supplementary Figures

Supplementary Tables
