# Supplementary material for: Prognostic value of prostaglandin I2 synthase and its correlation with tumor-infiltrating immune cells in lung cancer, ovarian cancer, and gastric cancer
Source: Aging (Albany NY). 2020 May 28;12(10):9658–85. doi: 10.18632/aging.103235 (PMC7288932; doi:10.18632/aging.103235)
Supplement: Supplementary Figures [file aging-12-103235-s002..pdf]

SUPPLEMENTARY FIGURES

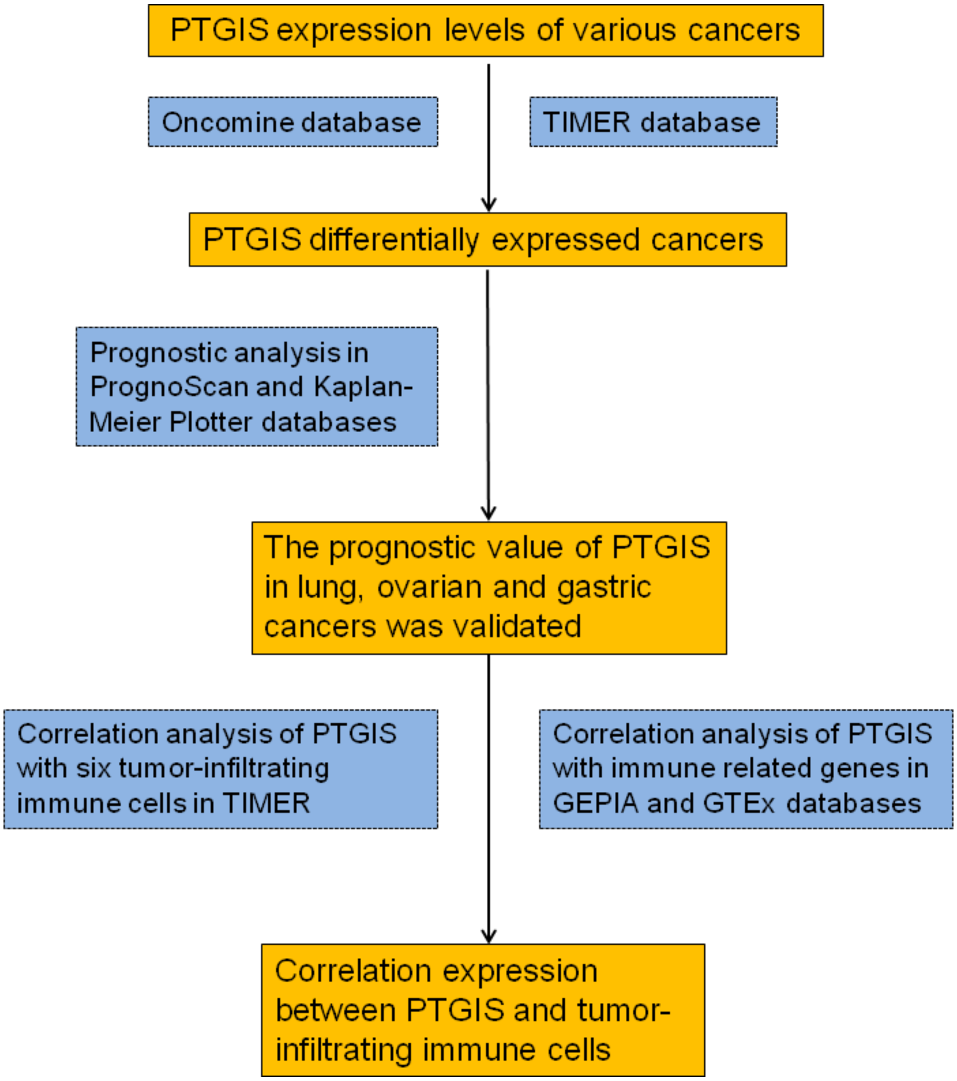

Supplementary Figure 1. Flow diagram.

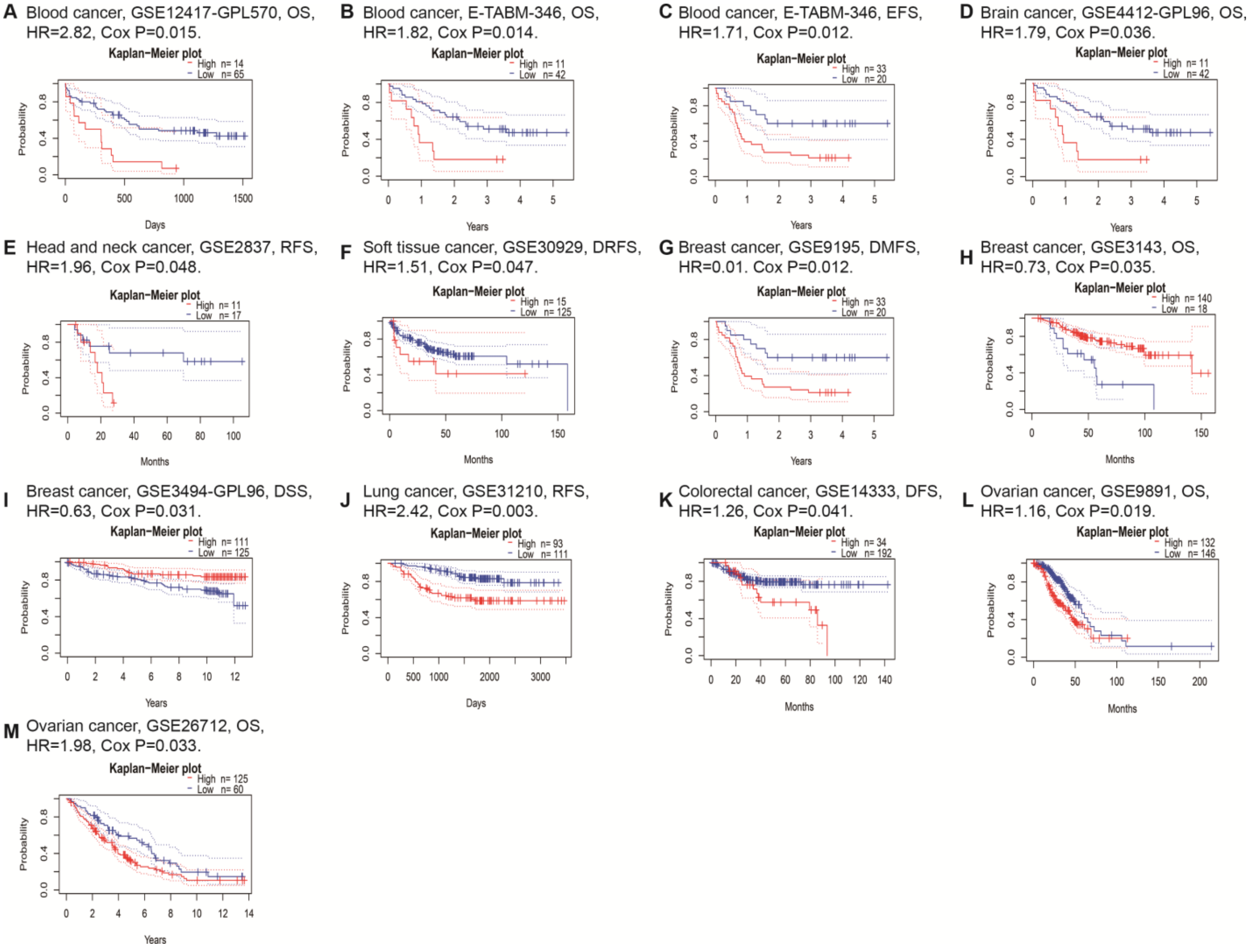

Supplementary Figure 2. Survival curves of high or low expression of PTGIS in different tumors from the PrognoScan database.

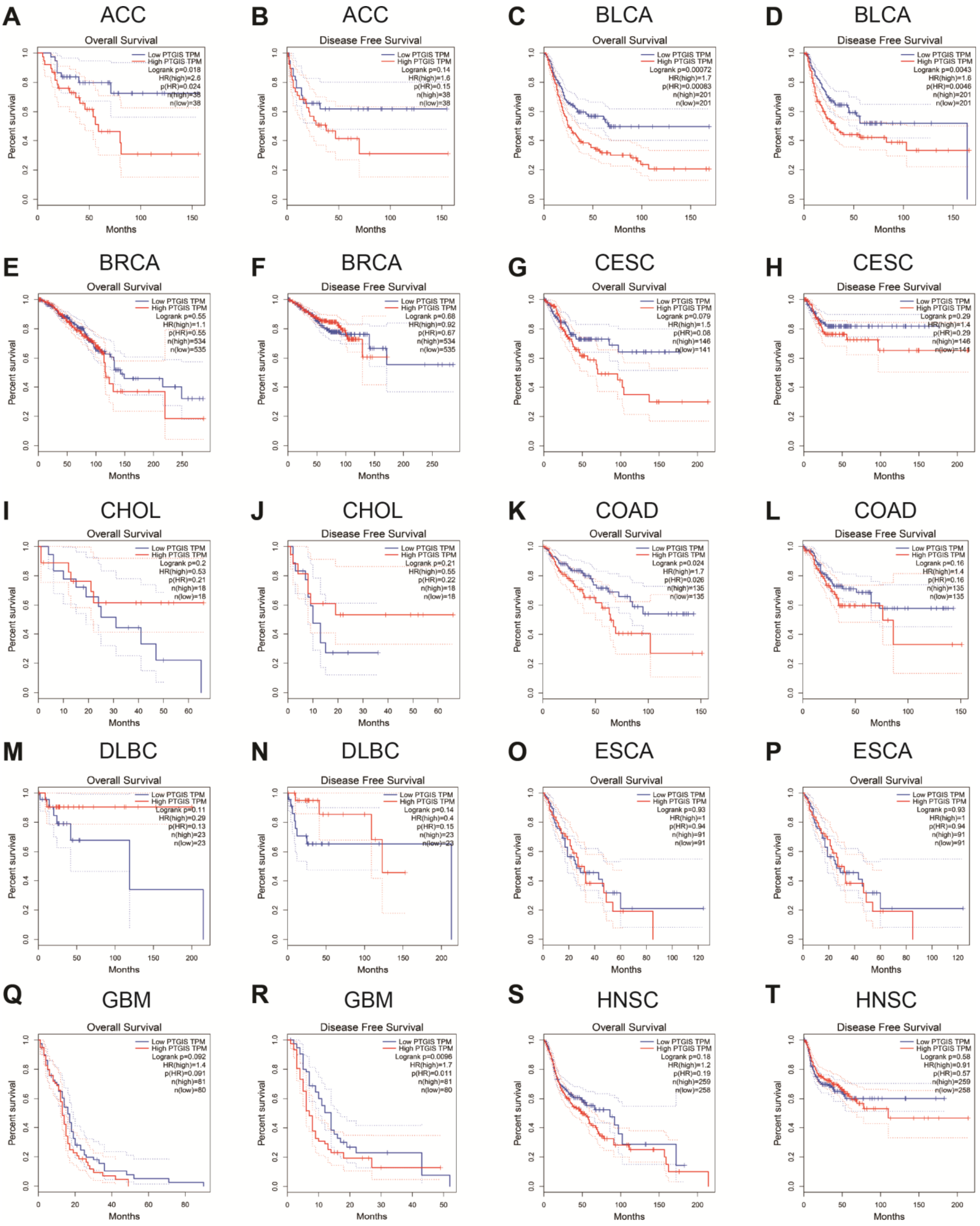
A ACC
Overall Survival
Low PTGIS TPM
High PTGIS TPM
Logrank p=0.018
HR(high)=2.6
p(HR)=0.024
n(high)=38
n(low)=38
B ACC
Disease Free Survival
Logrank p=0.14
HR(high)=1.6
p(HR)=0.15
n(high)=38
n(low)=38
C BLCA
Overall Survival
Logrank p=0.00072
HR(high)=1.7
p(HR)=0.00083
n(high)=201
n(low)=201
D BLCA
Disease Free Survival
Logrank p=0.0043
HR(high)=1.6
p(HR)=0.0046
n(high)=201
n(low)=201
E BRCA
Overall Survival
Logrank p=0.55
HR(high)=1.1
p(HR)=0.55
n(high)=534
n(low)=535
F BRCA
Disease Free Survival
Logrank p=0.68
HR(high)=0.92
p(HR)=0.67
n(high)=534
n(low)=535
G CESC
Overall Survival
Logrank p=0.079
HR(high)=1.5
p(HR)=0.08
n(high)=146
n(low)=141
H CESC
Disease Free Survival
Logrank p=0.29
HR(high)=1.4
p(HR)=0.29
n(high)=146
n(low)=141
I CHOL
Overall Survival
Logrank p=0.2
HR(high)=0.53
p(HR)=0.21
n(high)=18
n(low)=18
J CHOL
Disease Free Survival
Logrank p=0.21
HR(high)=0.55
p(HR)=0.22
n(high)=18
n(low)=18
K COAD
Overall Survival
Logrank p=0.024
HR(high)=1.7
p(HR)=0.026
n(high)=135
n(low)=135
L COAD
Disease Free Survival
Logrank p=0.16
HR(high)=1.4
p(HR)=0.16
n(high)=135
n(low)=135
M DLBC
Overall Survival
Logrank p=0.11
HR(high)=0.29
p(HR)=0.13
n(high)=23
n(low)=23
N DLBC
Disease Free Survival
Logrank p=0.14
HR(high)=0.4
p(HR)=0.15
n(high)=23
n(low)=23
O ESCA
Overall Survival
Logrank p=0.93
HR(high)=1
p(HR)=0.94
n(high)=91
n(low)=91
P ESCA
Overall Survival
Logrank p=0.93
HR(high)=1
p(HR)=0.94
n(high)=91
n(low)=91
Q GBM
Overall Survival
Logrank p=0.092
HR(high)=1.4
p(HR)=0.091
n(high)=81
n(low)=80
R GBM
Disease Free Survival
Logrank p=0.0096
HR(high)=1.7
p(HR)=0.011
n(high)=81
n(low)=80
S HNSC
Overall Survival
Logrank p=0.18
HR(high)=1.2
p(HR)=0.19
n(high)=259
n(low)=258
T HNSC
Disease Free Survival
Logrank p=0.58
HR(high)=0.91
p(HR)=0.57
n(high)=259
n(low)=258
Percent survival
Months

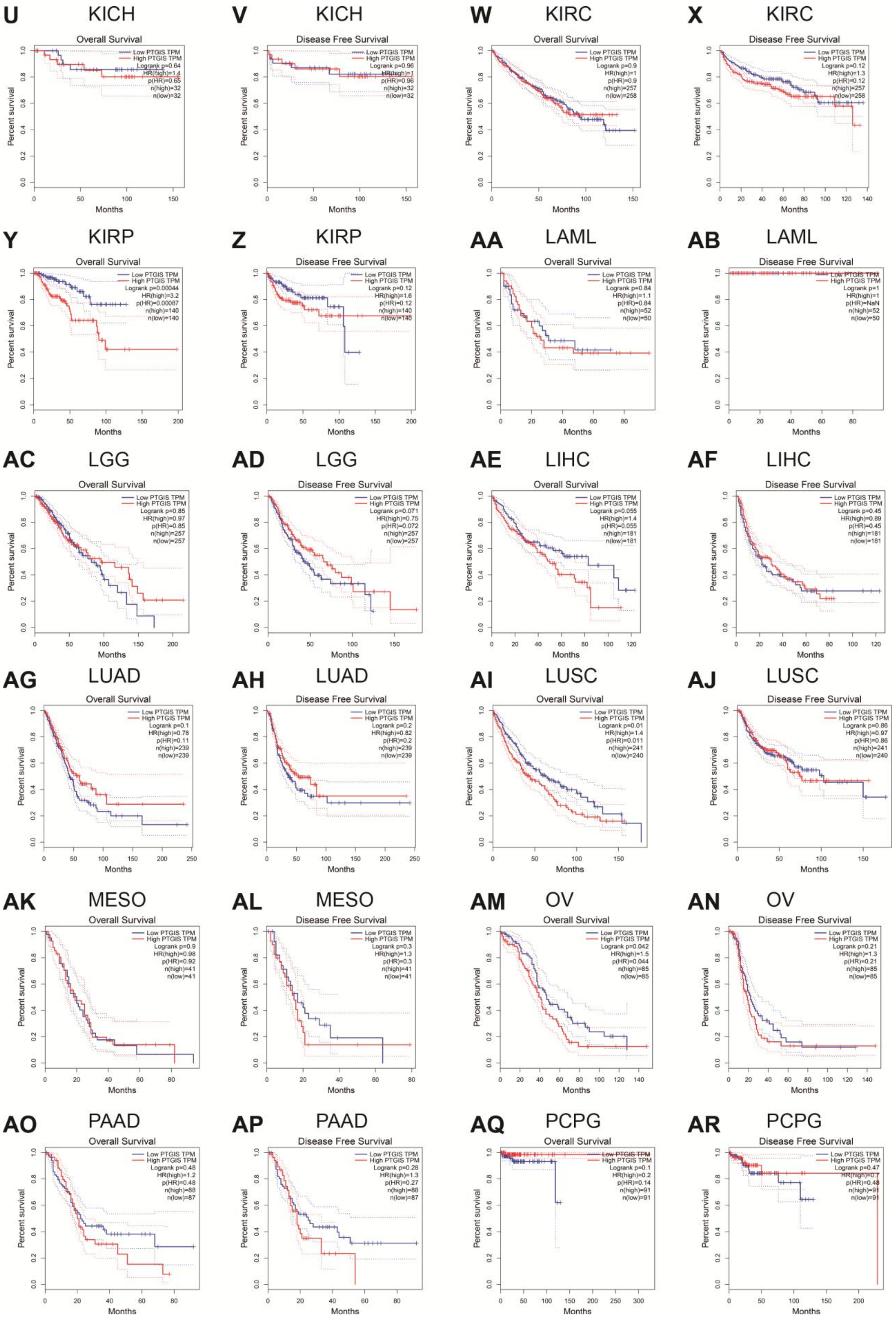

U
KICH
Overall Survival
V
KICH
Disease Free Survival
W
KIRC
Overall Survival
X
KIRC
Disease Free Survival
Y
KIRP
Overall Survival
Z
KIRP
Disease Free Survival
AA
LAML
Overall Survival
AB
LAML
Disease Free Survival
AC
LGG
Overall Survival
AD
LGG
Disease Free Survival
AE
LIHC
Overall Survival
AF
LIHC
Disease Free Survival
AG
LUAD
Overall Survival
AH
LUAD
Disease Free Survival
AI
LUSC
Overall Survival
AJ
LUSC
Disease Free Survival
AK
MESO
Overall Survival
AL
MESO
Disease Free Survival
AM
OV
Overall Survival
AN
OV
Disease Free Survival
AO
PAAD
Overall Survival
AP
PAAD
Disease Free Survival
AQ
PCPG
Overall Survival
AR
PCPG
Disease Free Survival
Percent survival
Months

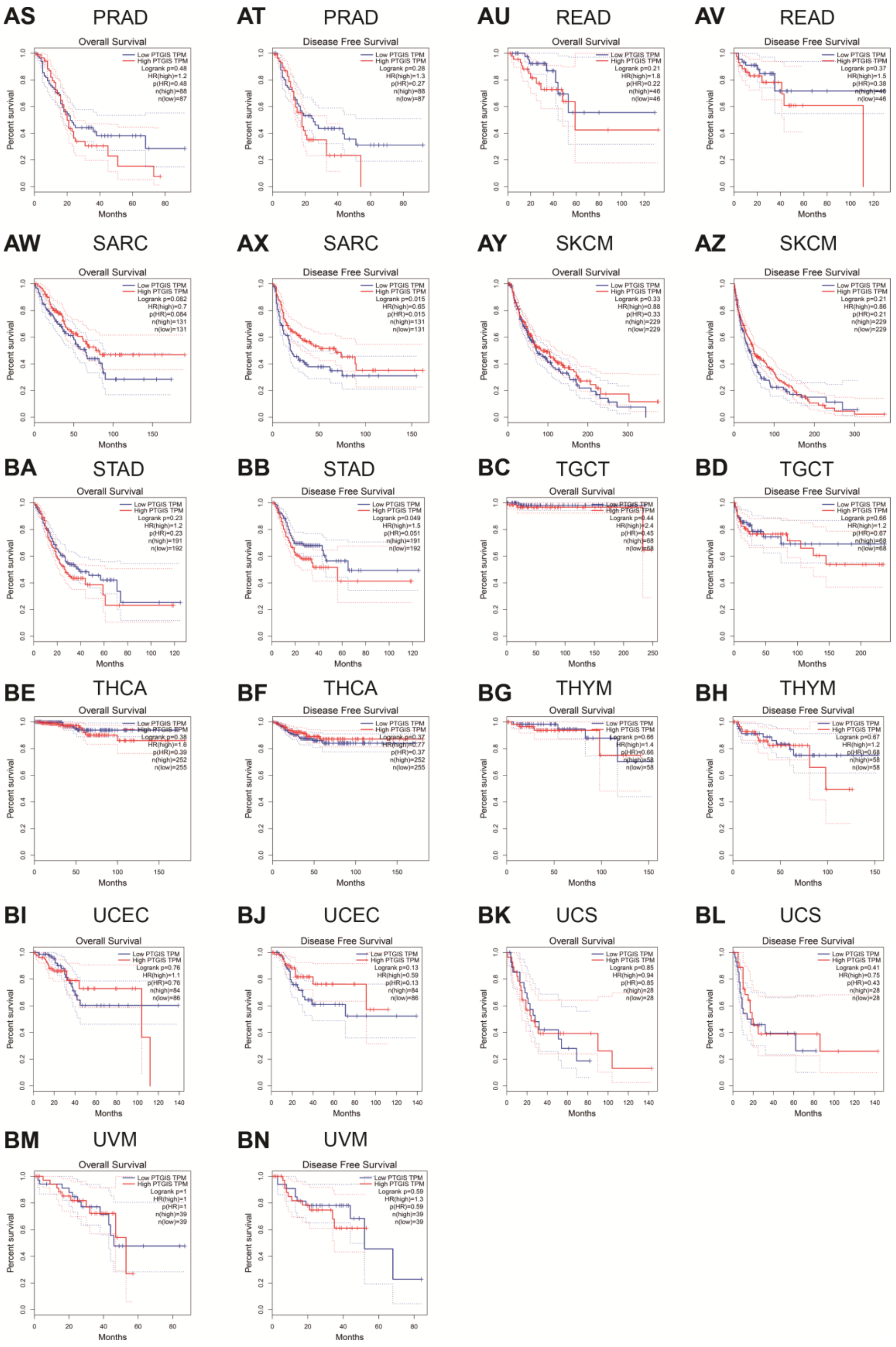

Supplementary Figure 3. Correlation of PTGIS expression with prognosis in diverse types of cancer.

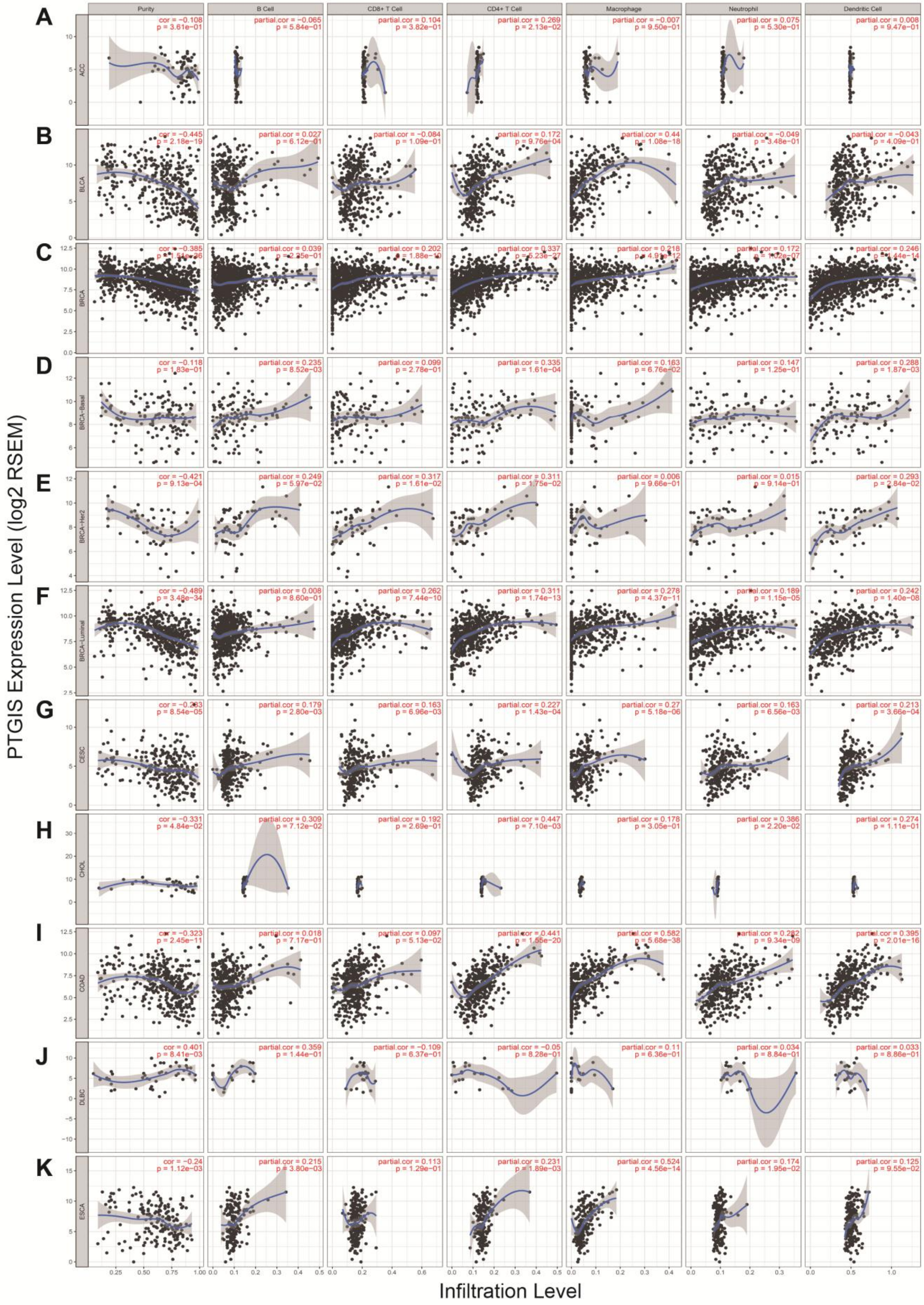
A
B
C
D
E
F
G
H
I
J
K
Purity
B Cell
CD8+ T Cell
CD4+ T Cell
Macrophage
Neutrophil
Dendritic Cell
ACC
BLCA
BRCA
BRCA-Basal
BRCA-Her2
BRCA-Luminal
CESC
CHOL
COAD
DLBC
ESCA
PTGIS Expression Level (log2 RSEM)
Infiltration Level

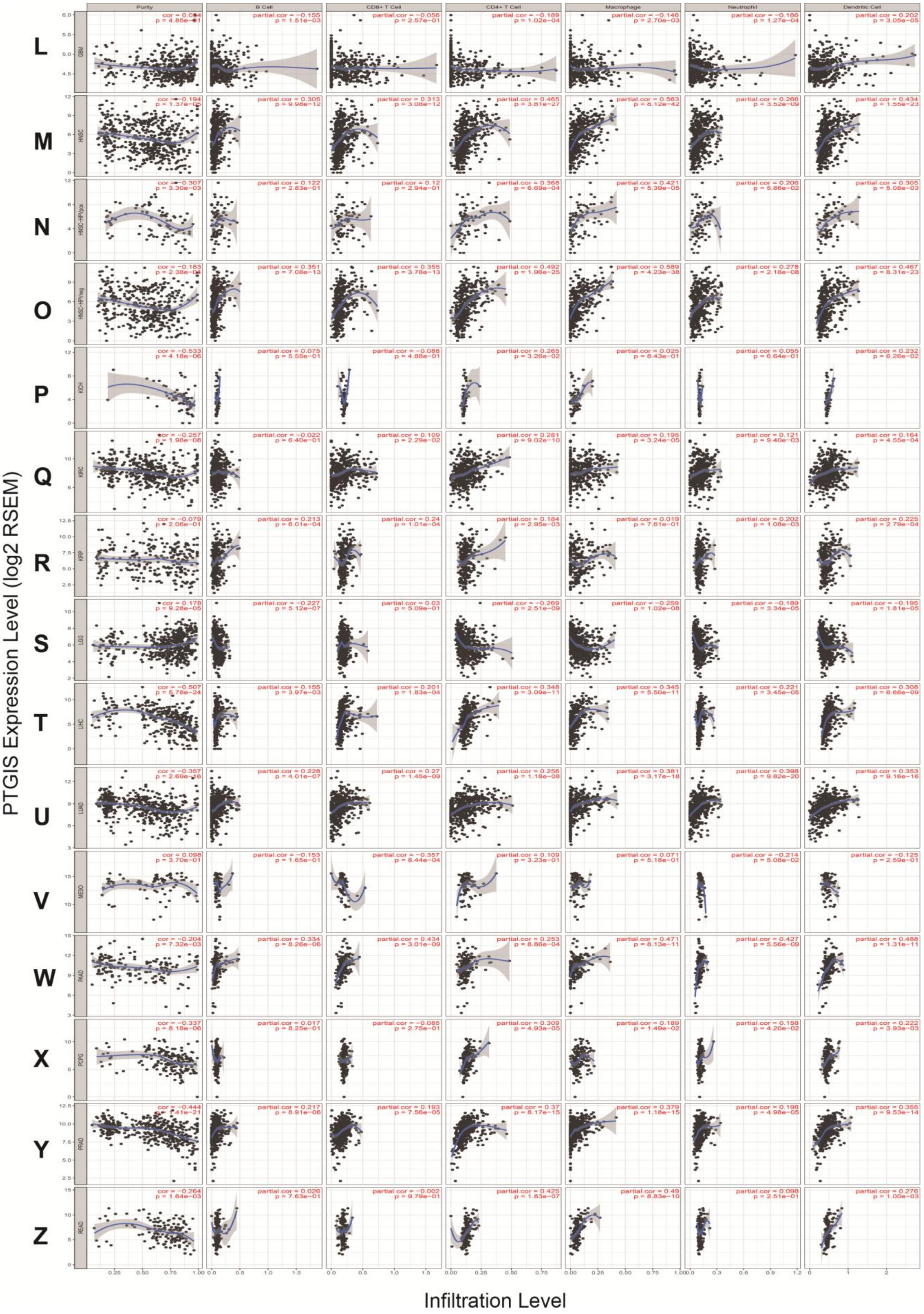

Purity
B Cell
CD8+ T Cell
CD4+ T Cell
Macrophage
Neutrophil
Dendritic Cell
L
M
N
O
P
Q
R
S
T
U
V
W
X
Y
Z
PTGIS Expression Level (log2 RSEM)
Infiltration Level

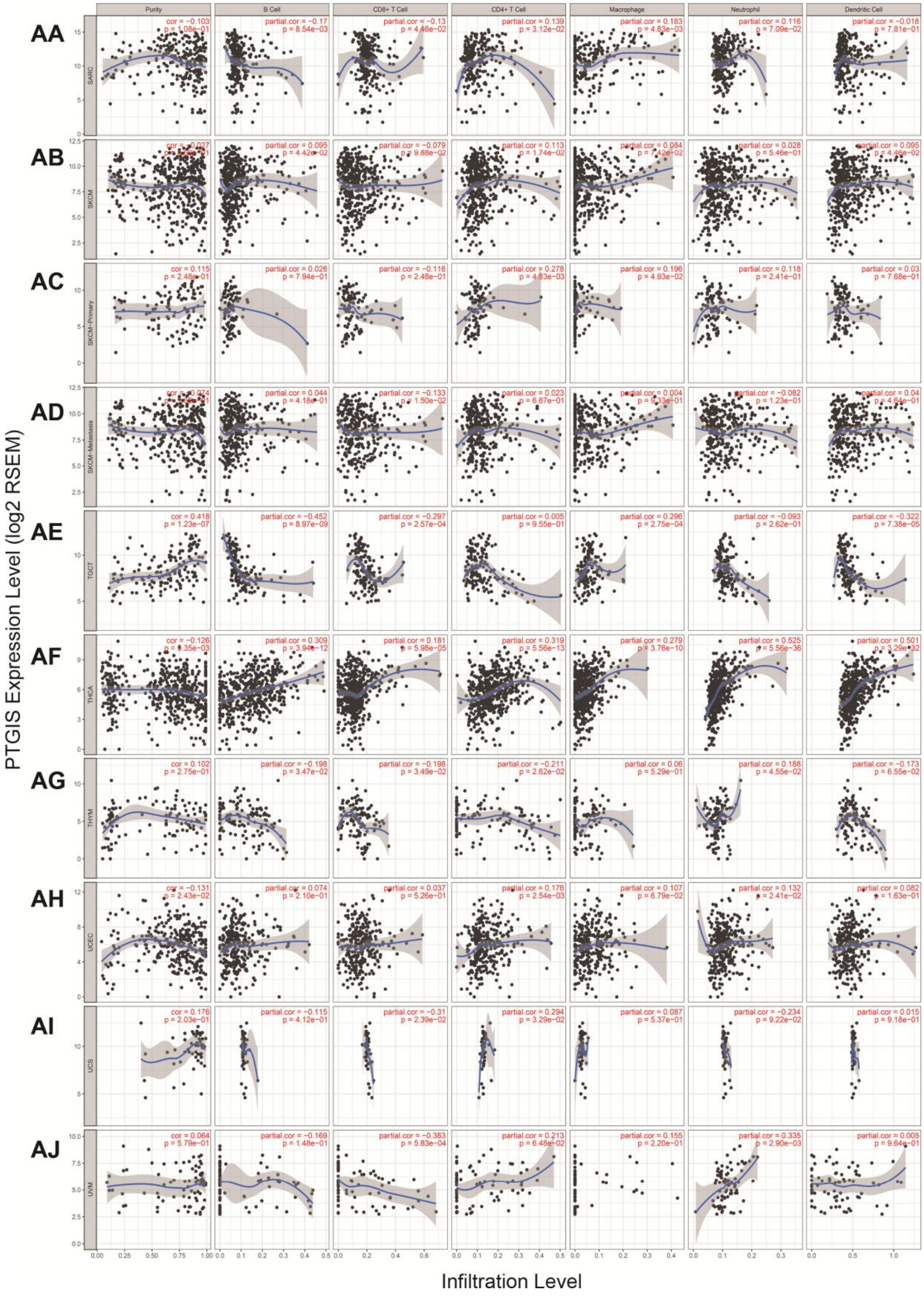

Supplementary Figure 4. Correlation of PTGIS expression with tumor-infiltrating immune cells in various types of cancers via the TIMER database.
