# Supplementary material for: Prognostic value of prostaglandin I2 synthase and its correlation with tumor-infiltrating immune cells in lung cancer, ovarian cancer, and gastric cancer
Source: Aging (Albany NY). 2020 May 28;12(10):9658–85. doi: 10.18632/aging.103235 (PMC7288932; doi:10.18632/aging.103235)
Supplement: Supplementary Tables [file aging-12-103235-s001..pdf]

SUPPLEMENTARY TABLES

Supplementary Table 1. PTGIS expression in cancers vs. normal tissue in Oncomine database.

Cancer	Cancer type	P-value	Fold change	Rank (%)	Sample	Reference (PMID)
Bladder	Superficial Bladder Cancer	8.01E-25	-31.339	1%	76	16432078
	Infiltrating Bladder Urothelial Carcinoma	7.30E-24	-8.600	1%	129	16432078
	Superficial Bladder Cancer	1.28E-15	-4.420	1%	194	20421545
	Infiltrating Bladder Urothelial Carcinoma	2.09E-07	-2.889	4%	130	20421545
	Superficial Bladder Cancer	7.13E-07	-3.141	4%	42	15173019
Breast	Invasive Breast Carcinoma Stroma	6.26E-14	6.187	7%	59	18438415
	Ductal Breast Carcinoma	1.39E-07	-2.352	1%	39	10963602
	Invasive Breast Carcinoma	1.88E-08	-3.227	4%	165	22522925
	Invasive Ductal and Invasive Lobular Breast Carcinoma	6.68E-30	-2.667	4%	234	22522925
	Invasive Lobular Breast Carcinoma	2.97E-29	-2.341	5%	292	22522925
	Medullary Breast Carcinoma	9.26E-12	-2.592	6%	176	22522925
	Invasive Ductal Breast Carcinoma	1.20E-47	-2.943	6%	1700	22522925
	Tubular Breast Carcinoma	1.25E-18	-2.339	8%	211	22522925
	Mucinous Breast Carcinoma	7.35E-13	-3.019	9%	190	22522925
	Invasive Ductal Breast Carcinoma	2.96E-21	-5.071	9%	450	TCGA
Cervical	Cervical Squamous Cell Carcinoma	1.55E-06	-2.400	4%	56	18506748
	Cervical Squamous Cell Carcinoma	1.23E-06	-3.521	3%	45	18191186
Colorectal	Cecum Adenocarcinoma	3.87E-10	-7.035	8%	44	TCGA
	Rectal Adenocarcinoma	9.26E-13	-7.478	10%	82	TCGA
Head and Neck	Tongue Squamous Cell Carcinoma	9.85E-07	-5.788	3%	57	19138406
Kidney	Papillary Renal Cell Carcinoma	5.43E-18	4.434	1%	34	16115910
	Renal Oncocytoma	2.65E-18	2.870	2%	35	16115910
	Chromophobe Renal Cell Carcinoma	3.86E-06	2.892	5%	29	16115910
	Clear Cell Sarcoma of the Kidney	1.45E-07	-13.915	1%	17	16299227
Leukemia	Chronic Lymphocytic Leukemia	6.88E-05	-2.242	6%	111	15459216
Liver	Cirrhosis	8.49E-13	2.758	5%	77	19098997
	Hepatocellular Carcinoma	5.22E-20	-3.308	2%	171	12058060
	Hepatocellular Carcinoma	2.84E-46	-2.562	3%	445	21159642
	Hepatocellular Carcinoma	2.91E-06	-2.407	6%	43	21159642
Lung	Lung Adenocarcinoma	2.73E-08	-2.500	3%	39	16314486
	Lung Carcinoid Tumor	1.06E-07	-58.003	6%	37	11707567
	Lung Adenocarcinoma	2.60E-17	-2.314	4%	116	22613842
	Lung Adenocarcinoma	6.11E-06	-2.780	8%	57	17540040
Melanoma	Cutaneous Melanoma	9.44E-05	2.148	1%	18	18442402
	Benign Melanocytic Skin Nevus	2.05E-06	-5.545	2%	25	16243793
	Cutaneous Melanoma	7.38E-09	-11.640	3%	52	16243793
Ovarian	Ovarian Serous Cystadenocarcinoma	3.00E-06	-6.836	3%	594	TCGA
	Ovarian Carcinoma	3.74E-07	-6.303	8%	195	18593951
	Ovarian Serous Adenocarcinoma	7.59E-07	-4.999	10%	45	19486012
Pancreatic	Pancreatic Ductal Adenocarcinoma	9.45E-05	2.826	1%	49	16053509
	Pancreatic Ductal Adenocarcinoma	3.20E-11	4.660	3%	78	19260470
Prostate	Prostate Carcinoma	6.69E-07	-2.467	5%	87	22722839
Sarcoma	Gastrointestinal Stromal Tumor	2.23E-13	9.876	1%	25	21447720
	Clear Cell Sarcoma of the Kidney	1.45E-07	-13.915	1%	17	16299227
Other	Pleural Malignant Mesothelioma	1.33E-06	3.368	2%	49	15920167
	Teratoma	1.05E-07	3.328	5%	20	16424014

Supplementary Table 2. Positive results associated with PTGIS expression in different cancers from Prognoscan database.

Cancer type	Dataset	Endpoint	N	Hazard ratio (95%CI)	Cox P-value
Blood cancer	GSE12417-GPL570	OS	79	2.82 [1.23 - 6.47]	0.015
	E-TABM-346	EFS	53	1.71 [1.12 - 2.59]	0.012
	E-TABM-346	OS	53	1.82 [1.13 - 2.94]	0.014
Brain cancer	GSE4412-GPL96	OS	74	1.79 [1.04 - 3.09]	0.036
Breast cancer	GSE3143	OS	158	0.73 [0.55 - 0.98]	0.035
	GSE9195	DMFS	77	0.01 [0.00 - 0.39]	0.012
	GSE1456-GPL96	DSS	159	0.60 [0.40 - 0.90]	0.013
	GSE1456-GPL96	OS	159	0.63 [0.44 - 0.90]	0.012
	GSE3494-GPL96	DSS	236	0.63 [0.42 - 0.96]	0.031
Colorectal cancer	GSE17536	DSS	177	1.34 [1.01 - 1.77]	0.042
	GSE17536	DFS	145	1.63 [1.15 - 2.30]	0.006
	GSE14333	DFS	226	1.26 [1.01 - 1.58]	0.041
Head and neck cancer	GSE2837	RFS	28	1.96 [1.01 - 3.83]	0.048
Lung cancer	GSE31210	RFS	204	2.42 [1.35 - 4.35]	0.003
	GSE14814	OS	90	7.50 [1.68 - 33.39]	0.008
	GSE14814	DFS	90	5.88 [1.05 - 33.10]	0.044
Ovarian cancer	GSE9891	OS	278	1.16 [1.02 - 1.32]	0.019
	GSE8841	OS	81	4.00 [1.29 - 12.42]	0.016
	GSE26712	DFS	185	1.90 [1.06 - 3.40]	0.031
	GSE26712	OS	185	1.98 [1.06 - 3.70]	0.033
Soft tissue cancer	GSE30929	DRFS	140	1.51 [1.00 - 2.28]	0.047

Abbreviation: OS Overall survival; DFS Disease free survival; EFS Event free survival; DMFS Distant metastasis free survival; RFS Relapse free survival; DSS Disease specific survival; CI Confidence interval

Supplementary Table 3. Correlation of PTGIS mRNA expression and clinicopathological factors in ovarian cancer by Kaplan-Meier plotter database.

Variables of ovarian cancer	Overall survival (n=1657)			Progression-free survival (n=1436)		
	N	Hazard ratio	P-value	N	Hazard ratio	P-value
**Histology**						
Endometroid	37	2.84(0.47-17.01)	0.2319	51	2.15(0.71-6.55)	0.1677
Serous	1207	1.26(1.07-1.48)	**0.0055**	1104	1.33(1.14-1.54)	**0.0002**
**Stage**						
1	74	3.39(0.74-15.51)	0.0940	96	2.48(0.69-8.91)	0.1498
2	61	2.39(0.51-11.23)	0.2574	67	2.34(0.9-6.09)	0.0721
3	1044	1.2(1.01-1.42)	**0.0398**	919	1.28(1.09-1.50)	**0.0025**
4	176	1.39(0.92-2.11)	0.1159	162	0.82(0.54-1.24)	0.3466
**Grade**						
1	56	2.44(0.9-6.59)	0.0698	37	4.93(1.61-15.08)	0.0020
2	324	1.41(1.03-1.92)	**0.0305**	256	1.96(1.41-2.72)	**4.60E-05**
3	1015	1.16(0.97-1.38)	0.0940	837	1.24(1.05-1.48)	0.0123
**TP53 mutation**						
Mutated	506	1.27(0.98-1.65)	0.0651	483	1.27(0.98-1.63)	0.0663
Wild type	94	2.2(1.27-3.8)	**0.0040**	84	1.57(0.87-2.83)	0.1291
**Debulk**						
Optimal	801	1.21(0.99-1.49)	0.0656	696	1.26(1.04-1.53)	**0.0181**
Suboptimal	536	1.26(1.03-1.54)	**0.0266**	459	0.80(0.65-0.99)	**0.0375**
**Chemotherapy**						
contains platin	1409	1.29(1.12-1.49)	**0.0003**	1259	1.23(1.08-1.40)	**0.0017**
contains Taxol	793	1.24(1.01-1.52)	**0.0369**	715	1.28(1.08-1.52)	**0.0041**
contains Taxol+platin	776	1.24(1.01-1.53)	**0.0404**	698	1.28(1.08-1.53)	**0.0049**
contains Avastin	50	2.08(0.72-6.02)	0.1693	50	1.75(0.83-3.70)	0.1390

contains Docetaxel	108	1.46(0.8-2.64)	0.2129	106	1.96(1.13-3.39)	**0.0141**
contains Gemcitabine	135	1.64(1.07-2.52)	**0.0230**	131	1.49(0.95-2.34)	0.0787
contains Paclitaxel	220	1.53(0.97-2.42)	0.0658	229	1.32(0.92-1.90)	0.1346
contains Topotecan	119	1.66(1.11-2.49)	**0.0133**	118	1.41(0.90-2.20)	0.1307

Bold values indicate P < 0.05.

Supplementary Table 4. Correlation of PTGIS mRNA expression and clinicopathological factors in gastric cancer by Kaplan-Meier plotter database.

Variables of gastric cancer	Overall survival		(n=882)	Progression-free survival		(n=646)
	N	Hazard ratio	P-value	N	Hazard ratio	P-value
**Gender**						
Female	236	1.13(0.98-1.31)	0.1003	201	2.07(1.40-3.04)	**0.0002**
Male	545	2.18(1.75-2.70)	**5.80E-13**	438	2.20(1.73-2.80)	**4.70E-11**
**Stage**						
2	140	2.17(1.17-4.02)	**0.0118**	131	1.58(0.84-2.95)	0.1499
3	305	2.39(1.63-3.50)	**4.00E-06**	186	1.84(1.25-2.69)	**0.0015**
4	148	1.48(1.00-2.20)	**0.0485**	141	1.38(0.93-2.04)	0.1114
**Stage T**						
2	241	1.60(1.03-2.50)	**0.0358**	239	1.51(1.00-2.29)	**0.0495**
3	204	2.48(1.63-3.77)	**1.30E-05**	204	1.81(1.23-2.67)	**0.0024**
4	38	1.82(0.72-4.62)	**0.1992**	39	2.13(0.95-4.76)	0.0605
**Stage N**						
0	74	2.43(0.99-5.93)	**0.0453**	72	2.10(0.88-5.01)	0.0868
1	225	2.19(1.44-3.32)	**0.0002**	222	2.00(1.35-2.97)	**0.0005**
2	121	3.12(1.95-4.98)	**5.60E-07**	125	2.33(1.50-3.61)	**1.00E-04**
3	76	1.70(0.99-2.94)	0.0538	76	1.75(1.01-3.02)	**0.0428**
1+2+3	422	2.08(1.57-2.74)	**1.50E-07**	423	1.76(1.37-2.28)	**1.10E-05**
**Stage M**						
0	444	2.03(1.51-2.72)	**1.80E-06**	443	1.64(1.26-2.14)	**0.0002**
1	56	1.87(1.03-3.41)	**0.0372**	56	0.70(0.36-1.34)	0.2771
**HER2 status**						
negative	532	2.06(1.62-2.62)	**1.50E-09**	408	1.88(1.44-2.45)	**2.00E-06**
positive	344	1.98(1.50-2.61)	**7.30E-07**	233	2.43(1.76-3.37)	**3.20E-08**
**Lauren classification**						
Intestinal	320	2.33(1.70-3.21)	**8.10E-08**	263	1.81(1.27-2.57)	**0.0009**
Diffuse	241	1.75(1.22-2.52)	**0.0022**	231	1.54(1.09-2.17)	**0.0134**
**Differentiation**						
poorly	165	0.76(0.48-1.20)	0.2404	121	0.66(0.38-1.12)	0.1192
moderately	67	3.56(1.22-10.43)	**0.0145**	67	3.10(1.18-8.15)	**0.0167**

Bold values indicate P < 0.05.

Supplementary Table 5. Correlation analysis between PTGIS and relate genes and markers of innate immunity cells in TIMER.

Description	Gene markers	LUSC				OV				STAD			
		Purity		None		Purity		None		Purity		None	
		Cor	P	Cor	P	Cor	P	Cor	P	Cor	P	Cor	P
Monocyte	CD14	0.387	***	0.468	***	-0.032	0.618	0.357	***	0.369	***	0.368	***
	CD86	0.372	***	0.452	***	-0.077	0.226	0.289	***	0.303	***	0.300	***
	CD16(FCGR3A)	0.433	***	0.496	***	0.023	0.720	0.354	***	0.274	***	0.269	***
TAM	CD68	0.302	***	0.392	***	-0.029	0.653	0.329	***	0.123	0.016	0.136	*
	CCL2	0.456	***	0.503	***	0.034	0.590	0.325	***	0.482	***	0.486	***
	CCL5	0.188	***	0.279	***	-0.061	0.337	0.242	***	0.217	***	0.219	***
M1 Macrophage	INOS (NOS2)	0.178	***	0.168	**	0.093	0.145	0.172	*	-0.137	*	-0.123	0.012
	CXCL10	0.111	0.015	0.184	***	-0.235	**	-0.034	0.556	-0.022	0.671	-0.001	0.982
	TNF-α (TNF)	0.048	0.291	0.152	**	-0.015	0.613	0.080	0.167	-0.067	0.194	-0.060	0.222
M2 Macrophage	CD206(MRC1)	0.442	***	0.504	***	0.157	0.013	0.421	***	0.368	***	0.367	***
	CD163	0.471	***	0.534	***	-0.092	0.147	0.398	***	0.367	***	0.366	***
	IL10	0.342	***	0.410	***	0.211	**	0.414	***	0.359	***	0.346	***
Neutrophils	CD66b (CEACAM8)	0.134	*	0.158	**	0.122	0.055	0.074	0.198	-0.028	0.587	-0.019	0.702
	CD11b (ITGAM)	0.436	***	0.502	***	-0.001	0.992	0.341	***	0.416	***	0.410	***
	CCR7	0.343	***	0.429	***	0.058	0.359	0.301	***	0.427	***	0.413	***
	CD15(FUT4)	0.219	***	0.247	***	0.109	0.086	0.197	**	-0.245	***	-0.239	***
Natural killer cell	KIR2DL1	0.092	0.046	0.139	*	0.041	0.519	0.132	0.021	0.087	0.090	0.105	0.032
	KIR2DL3	0.177	**	0.210	***	-0.026	0.687	0.064	0.266	0.025	0.630	0.067	0.171
	KIR2DL4	0.055	0.235	0.114	0.011	-0.236	**	0.007	0.906	-0.157	*	-0.141	*
	KIR3DL1	0.213	***	0.267	***	0.036	0.574	0.180	*	0.064	0.216	0.086	0.082
	KIR3DL2	0.130	*	0.186	***	0.002	0.975	0.141	0.014	0.114	0.027	0.138	*
	KIR3DL3	0.071	0.123	0.095	0.039	0.034	0.593	0.059	0.310	-0.135	*	-0.116	0.018
	KIR2DS4	0.173	**	0.207	***	0.061	0.336	0.149	**	0.003	0.958	0.013	0.799
Dendritic cell	HLA-DPB1	0.399	***	0.476	***	-0.142	0.025	0.169	**	0.247	***	0.249	***
	HLA-DQB1	0.253	***	0.340	***	-0.052	0.413	0.161	**	0.109	0.034	0.120	0.015
	HLA-DRA	0.365	***	0.442	***	-0.166	*	0.104	0.071	0.127	0.013	0.134	*
	HLA-DPA1	0.407	***	0.479	***	-0.148	0.019	0.136	0.018	0.174	**	0.181	**
	BDCA-1(CD1C)	0.263	***	0.372	***	0.014	0.820	0.245	***	0.528	***	0.502	***
	BDCA-4(NRP1)	0.350	***	0.427	***	0.164	*	0.412	***	0.551	***	0.551	***
	CD11c (ITGAX)	0.399	***	0.479	***	-0.003	0.956	0.306	***	0.324	***	0.335	***
	NKp46(NCR1)	0.211	***	0.263	***	-0.003	0.962	0.166	**	0.171	**	0.188	**

LUSC, lung squamous cell carcinoma; OV, ovarian serous cystadenocarcinoma; STAD, stomach adenocarcinoma; TAM, tumor-associated macrophage; Cor, R value of Spearman's correlation; None, correlation without adjustment. Purity, correlation adjusted by purity. *P < 0.01; **P < 0.001; ***P < 0.0001.

Supplementary Table 6. Correlation analysis between PTGIS and relate genes and markers of adaptive immunity cells in TIMER.

Description	Gene markers	LUSC				OV				STAD			
		Purity		None		Purity		None		Purity		None	
		Cor	P	Cor	P	Cor	P	Cor	P	Cor	P	Cor	P
CD8+ T cell	CD8A	0.275	***	0.346	***	0.007	0.918	0.284	***	0.280	***	0.286	***
	CD8B	0.297	***	0.335	***	0.002	0.970	0.222	**	0.220	***	0.215	***
T cell (general)	CD3D	0.239	***	0.339	***	-0.031	0.631	0.278	***	0.188	**	0.189	**
	CD3E	0.318	***	0.409	***	0.000	0.999	0.309	***	0.242	***	0.227	***
	CD2	0.307	***	0.359	***	-0.014	0.831	0.293	***	0.254	***	0.247	***
B cell	CD19	0.344	***	0.438	***	-0.025	0.699	-0.033	0.573	0.365	***	0.348	***
	CD20(MS4A1)	0.334	***	0.428	***	0.164	*	0.326	***	0.408	***	0.401	***
	CD138(SDC1)	-0.171	**	-0.152	**	0.191	*	0.320	***	-0.290	***	-0.297	***
	CD23(FCER2)	0.343	***	0.429	***	0.213	**	0.313	***	0.405	***	0.392	***
Th1	T-bet (TBX21)	0.276	***	0.361	***	-0.046	0.467	0.269	***	0.255	***	0.252	***
	STAT4	0.311	***	0.396	***	0.048	0.446	0.289	***	0.328	***	0.319	***
	STAT1	0.046	0.318	0.119	*	-0.230	**	-0.118	0.041	-0.070	0.172	-0.064	0.196
	IFN-γ (IFNG)	0.076	0.098	0.134	*	-0.125	0.049	0.125	0.029	-0.092	0.074	-0.093	0.057
	TNF-α (TNF)	0.048	0.291	0.152	**	-0.015	0.613	0.080	0.167	-0.067	0.194	-0.060	0.222
Th2	GATA3	0.215	***	0.288	***	0.004	0.946	0.253	***	0.384	***	0.375	***
	STAT6	-0.063	0.170	-0.033	0.462	-0.100	0.115	-0.110	0.055	0.122	0.018	0.129	*
	STAT5A	0.334	***	0.414	***	0.027	0.677	0.136	0.018	0.360	***	0.350	***
	IL13	0.177	***	0.223	***	0.053	0.401	0.072	0.211	0.131	0.010	0.130	*
Tfh	BCL6	0.019	0.685	0.000	0.998	0.000	0.997	-0.027	0.642	0.530	***	0.517	***
	IL21	0.156	**	0.213	***	-0.114	0.073	-0.082	0.154	0.052	0.312	0.049	0.318
	CD278(ICOS)	0.280	***	0.374	***	-0.033	0.606	0.235	***	0.130	0.012	0.132	*
	CXCL13	0.197	***	0.293	***	-0.026	0.680	0.199	**	0.243	***	0.236	***
Th17	STAT3	0.184	***	0.232	***	0.038	0.549	0.222	**	0.363	***	0.365	***
	IL17A	0.038	0.405	0.074	0.097	-0.105	0.097	0.042	0.470	-0.261	***	-0.272	***
Treg	FOXP3	0.350	***	0.429	***	-0.024	0.711	0.240	***	0.241	***	0.244	***
	CCR8	0.381	***	0.453	***	-0.033	0.604	0.130	0.024	0.344	***	0.345	***
	STAT5B	0.262	***	0.264	***	0.269	***	0.275	***	0.603	***	0.608	***
	TGFβ (TGFB1)	0.084	0.067	0.181	***	0.169	*	0.472	***	0.527	***	0.528	***
	CD25(IL2RA)	0.346	***	0.429	***	0.200	*	0.450	***	0.187	**	0.197	***
T cell exhaustion	PD-1 (PDCD1)	0.271	***	0.356	***	-0.106	0.096	0.200	**	0.147	*	0.158	*
	CTLA4	0.253	***	0.349	***	-0.044	0.490	0.240	***	0.088	0.087	0.092	0.062
	LAG3	0.128	*	0.206	***	-0.169	*	0.051	0.373	0.075	0.145	0.080	0.103
	TIM-3 (HAVCR2)	0.390	***	0.464	***	-0.036	0.572	0.336	***	0.291	***	0.294	***
	GZMB	0.144	*	0.236	***	-0.068	0.282	0.203	**	-0.089	0.085	-0.064	0.194

LUSC, lung squamous cell carcinoma; OV, ovarian serous cystadenocarcinoma; STAD, stomach adenocarcinoma; Th, T helper cell; Tfh, Follicular helper T cell; Treg, regulatory T cell; Cor, R value of Spearman's correlation; None, correlation without adjustment. Purity, correlation adjusted by purity. *P < 0.01; **P < 0.001; ***P < 0.0001.

Supplementary Table 7. Correlation analysis between PTGIS and immune relate genes of monocyte and macrophages in GEPIA.

Description	Gene markers	LUSC				OV				STAD			
		Normal		Tumor		Normal		Tumor		Normal		Tumor	
		R	P	R	P	R	P	R	P	R	P	R	P
**Monocyte**	CD14	0.011	0.86	0.49	***	0.46	***	0.41	***	0.67	***	0.40	***
	CD86	-0.011	0.86	0.46	***	0.41	***	0.36	***	0.49	***	0.33	***
	CD16(FCGR3A)	6E-04	0.99	0.52	***	0.42	***	0.43	***	0.51	***	0.30	***
**TAM**	CD68	-0.11	0.052	0.42	***	0.39	**	0.4	***	0.44	***	0.19	***
	CCL2	0.085	0.150	0.51	***	0.43	***	0.31	***	0.66	***	0.51	***
	CCL5	0.22	**	0.27	***	0.21	0.044	0.2	***	-0.27	**	0.23	***
**M1 Macrophage**	INOS (NOS2)	0.19	*	0.18	***	-0.084	0.440	0.36	***	0.63	***	-0.086	0.081
	CXCL10	0.22	**	0.17	**	-0.18	0.095	0.018	0.710	0.03	0.690	0.023	0.640
	TNF-α (TNF)	0.039	0.51	0.15	**	0.13	0.240	0.16	0.001	0.27	**	-0.007	0.890
**M2 Macrophage**	CD206(MRC1)	-0.06	0.33	0.53	***	0.37	**	0.51	***	0.67	***	0.40	***
	CD163	-0.13	0.033	0.53	***	0.32	*	0.36	***	0.60	***	0.37	***
	IL10	0.029	0.620	0.42	***	0.44	***	0.49	***	0.62	***	0.38	***

LUSC, lung squamous cell carcinoma; OV, ovarian serous cystadenocarcinoma; STAD, stomach adenocarcinoma. TAM, Tumor-associated macrophages. Tumor, correlation analysis in tumor tissue of TCGA. Normal, correlation analysis in normal tissue of GTEx. *P < 0.01; **P < 0.001; ***P < 0.0001.
